# What matters to people with chronic conditions when accessing care in Australian general practice? A qualitative study of patient, carer, and provider perspectives

**DOI:** 10.1186/s12875-019-0973-0

**Published:** 2019-06-10

**Authors:** Hyun Jung Song, Sarah Dennis, Jean-Frédéric Levesque, Mark Fort Harris

**Affiliations:** 1 Centre for Primary Health Care and Equity, University of New South Wales, Sydney, Australia; 2 Faculty of Health Sciences, University of Sydney, Sydney, Australia; 3 Ingham Institute for Applied Medical Research, Liverpool, Australia; 4 Agency for Clinical Innovation, Chatswood, New South Wales Australia

**Keywords:** Patient experience, Access to care, Australian general practice, Patient and carer perspectives, Provider perspectives, Qualitative methods, Patient surveys

## Abstract

**Background:**

Research underpinning the patient experience of people with chronic conditions in Australian general practice is not well developed. We aimed to ascertain the perspectives of key stakeholders on aspects of patient experience, more specifically with regards to accessing general practice in Australia.

**Methods:**

Using a qualitative design, semi-structured interviews were conducted by telephone and face-to-face with people living with one or more chronic conditions, informal carers, and primary care providers between October 2016 and October 2017. Participants were recruited and selected from three demographically representative primary health networks across Sydney, Australia. Interview transcripts and researcher’s reflective fieldnotes were coded and analyzed for key themes of access. Analysis and interpretation of data were guided by Levesque’s model of access, a conceptual framework to evaluate access broadly and from corresponding patient- and provider-side dimensions.

**Results:**

A total of 40 interviews were included in the analysis. Most participants had attended their general practices for 10 years or more and had regular primary care providers. People with chronic conditions reported access barriers predominantly in their *ability to reach* services, which were related to illness-related disabilities (limited mobility, chronic pain, fatigue, frailty) and limitations in the *availability and accommodation* of health services to address patient preferences (unavailability of after-hours services, lack of alternative modes of service delivery). While cost was not a major barrier, we found a lack of clarity in the factors that determined providers’ decisions to waive or reduce costs for some patients and not others.

**Conclusions:**

People managing chronic conditions with a long-term primary care provider experienced access barriers in general practice, particularly in their ability to physically reach care and to do so on a timely basis. This study has important policy and practice implications, as it highlights patients’ experiences of accessing care and possible areas for improvement to appropriately respond to these experiences. Themes identified may be useful in the design of a patient experience survey tool specific to this population. While it incorporates perspectives from patients, carers and providers, this study could be further strengthened by including perspectives from culturally and linguistically underrepresented patient groups and more carers.

**Electronic supplementary material:**

The online version of this article (10.1186/s12875-019-0973-0) contains supplementary material, which is available to authorized users.

## Background

Patient feedback on their experience of care is one of the core quality dimensions in the World Health Organization (WHO) framework for health system performance and is a widely recognized promoter of patient-centered care [1, 2]. Understanding how patients experience the health care system can provide useful insight into how they observe, interact with and are impacted by the health care environment, and highlight specific areas for improvement [3–5]. In order to measure and respond to patient experiences in a meaningful way, it is important to know about the factors that influence these experiences within the context of a particular health care setting and population [6].

Chronic health conditions affect more than half of the Australian adult population and are managed in the majority of cases during general practice encounters [7]. Chronic care management in Australia is primarily carried out by a team of professionals including the general practitioner (GP), practice nurses (PNs), and allied health professionals, working together in mainly privately-owned group practices [8–11]. General practice staff play a multitude of crucial roles including GP referrals to allied health and specialist service providers [11, 12], care planning and coordination [13, 14], continuous monitoring of patient needs, and delivering self-management support and education [10]. GPs can also enable more affordable services to patients by directly billing services to Medicare (Australia’s tax-financed public insurance scheme) in a practice known as bulk-billing [11], which minimizes or eliminates individual copayments. Through a fee-for-services system, GPs in Australia are primarily remunerated through government rebates of services listed on the Medicare Benefits Schedule (MBS). Since 1999, that list has included the preparation and review of General Practice Management Plans (or “Care Plans”) and Team Care Arrangements (TCAs), which implement structured, personalized planning and coordination of multidisciplinary care for people with chronic or terminal conditions [15]. These GP-managed chronic disease management plans can also provide patients with Medicare subsidies for services that otherwise incur out-of-pocket fees, especially by allied health professionals.

General practice plays an important and multifaceted role in the care and management of people with chronic conditions in Australia. However, the factors that influence patient experience in Australian general practice have not been explored in depth. Within this context, the literature tends to be population or condition-specific [16–19] and one-sided (e.g. capturing patient or provider views only) [20–22]. Furthermore, despite the significant contribution of carers and family to the patient’s care and wellbeing [2, 23], their views are seldom included in academic research or practice improvement. This evidence gap needs to be addressed particularly for those who frequently access care at this level.

This qualitative study thus aims to understand the perspectives of people living with chronic conditions, their carers, and primary care providers about aspects of their experience, particularly in relation to accessing Australian general practice.

## Methods

Qualitative research methods were used. A phenomenological approach was used as it aims to understand people’s experience of reality and thus was well suited for the purpose of our study [24]. Semi-structured interviews were conducted over 12 months from October 2016 to October 2017. The interviews were conducted either face to face or by telephone with two main participant groups: (i) primary care providers, and (ii) people living with one or more chronic conditions and their carers.

### Participants

Participants were recruited from the geographical areas represented by three Primary Health Networks (PHNs) in Sydney, Australia. These PHNs were chosen for their demographic and geographic diversity, in order to capture a wide range of participant experiences and backgrounds reflecting cultural, linguistic, socioeconomic and geographic diversity.

We aimed to recruit up to 22 participants in each participant group (patient/carers and providers) (max total *n* = 44), to ensure representativeness of the sample. Participants were recruited through a purposive sampling.

### Primary care providers

Primary care providers were eligible to participate if they were practicing as a GP or practice nurse (PNs) in a general practice in one of the participating PHNs. GPs and PNs were recruited through a snowball approach from practices known to the researchers and those listed in directories provided by the PHNs, including those who had previously participated in one or more research projects or quality improvement programs. They were sent or faxed an invitation letter. Wherever possible, practitioners who specified their experience or interest in chronic disease management were targeted. Further snowball sampling of participants was conducted through referrals from interviewees.

### People with chronic conditions and carers

The study was advertised through Health Consumers NSW website and its social media accounts, targeting people with one or more chronic medical conditions and their primary carers. Patients were eligible for the study if they had a chronic condition defined as one that persisted 6 months or more after the diagnosis or identification of long term condition [25]. Patients were not excluded if they had not been given an official diagnosis for their condition. While rare conditions have been defined as those that have a population prevalence of less than 1 in 2000 [26, 27], those who self-reported as having conditions that were rare, unknown or not well understood by the medical community were included in the study. If a patient had a severe cognitive or psychological impairment that inhibited their ability to autonomously participate in the interviews, their experiences were captured through interviews with their primary carer instead. In such cases, interview questions were directed at capturing the patient and carer’s experiences of navigating care in general practice together. While we did not use any specific criterion for carers in this study, for most people with serious chronic conditions, carers often tend to be family or friends of the patient providing care and self-management support to patients in an informal, unpaid role [28]. Participants were excluded if their main provider was not based in one of the three participating PHNs (as per the conditions of ethics approval). A snowball sampling strategy was used to complete the recruitment of patients until thematic saturation as well as to ensure representation of patients of particular backgrounds (e.g. rare disease)), and included recruitment through patient support or advocacy organizations.

### Data collection

One of the researchers (HJS) conducted all the interviews with the providers in their office, each lasting approximately 20–60 min. All of the patient and carer interviews, lasting between 15 and 40 min, were conducted by telephone for two reasons: firstly, to ensure privacy and allow participants to speak freely without social pressure (by their primary care providers); and secondly, due to distance [29]. No repeat or follow-up interviews were conducted with the participants; however, they were invited to provide any additional, relevant responses that were not discussed during the interviews via email or phone post-interview.

The semi-structured interview guides were formulated and informed by the primary research question to gather (i) descriptive information about the patient experience (a walkthrough of a typical visit ‘from booking the appointment to leaving the practice’, as well as events that took place between visits) as well as (ii) participant perspectives on important aspects of patient experience and quality improvement in general practice. The guides were developed in collaboration with the research team (HJS, MH, SD, JFL). While separate interview guides were used for the patient/carer and provider interviews, the questions were designed to mirror each other and gather similar information from the two different perspectives (Additional files 1 and 2). They were pilot tested for comprehensibility and adapted appropriately with staff from the Centre for Primary Health Care and Equity, UNSW Australia, who fit the inclusion criteria for participants (e.g. person living with one or more chronic condition, and practicing GP in the Sydney area).

All interviews were audio-recorded and transcribed verbatim. The interviewer (HJS) also made field notes during the interviews. Once transcribed the transcripts were read and edited for clarity and completion using the notes and recordings. Transcripts were not returned to participants for comments or corrections.

### Coding framework and thematic analysis

**Fig. 1:**
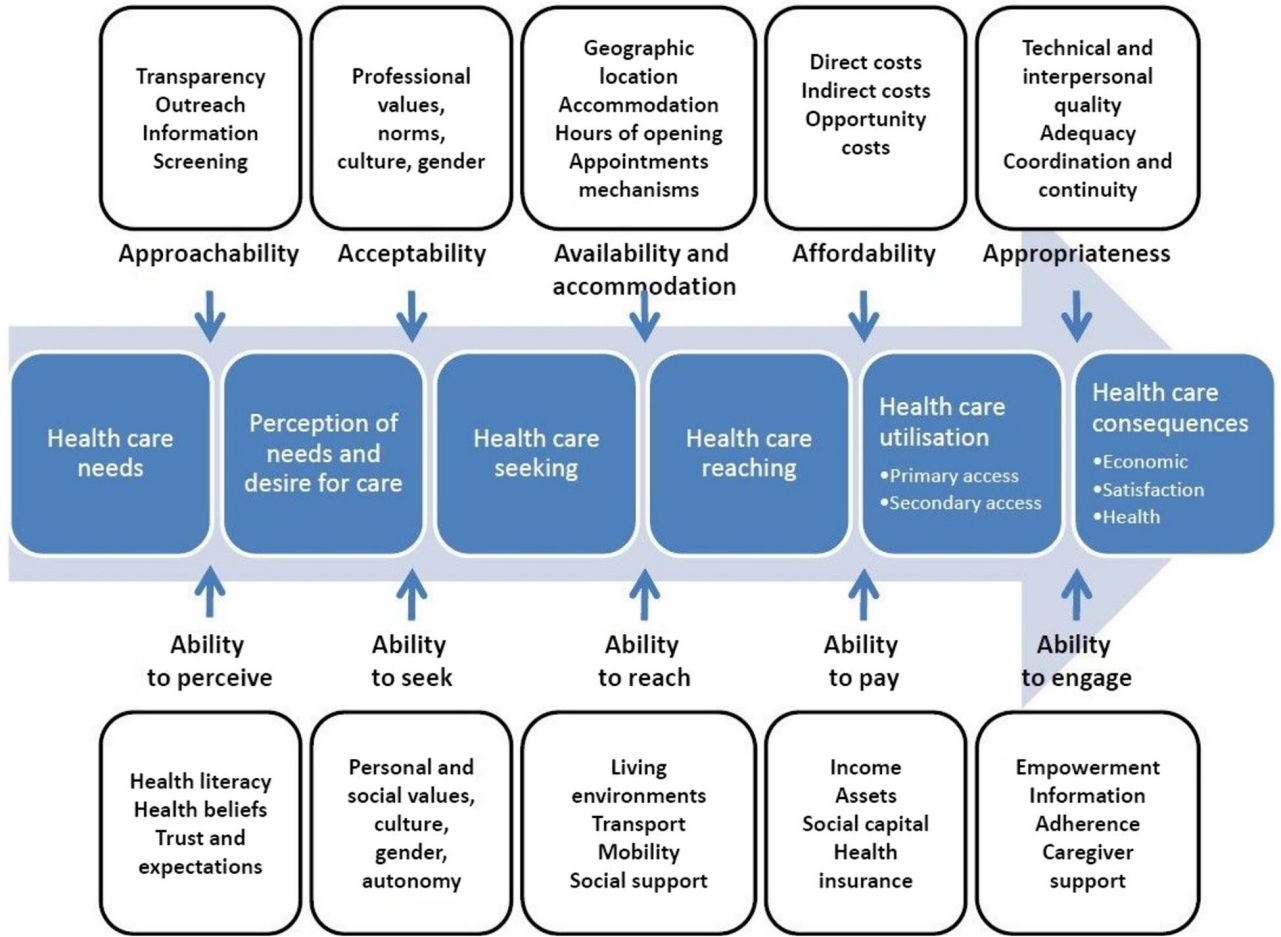
Levesque's model of access to heath care

A sample of three transcripts were reviewed collaboratively by three researchers (HJS, MFH, SD) to familiarize themselves with the data and to discuss differences in coding and interpretation. The Levesque’s model of health care access [30] was selected as the coding framework for two reasons. Firstly, the model explores access to care comprehensively within five sequential ‘domains’ or timepoints in the patient’s care journey: 1) perceiving the need for care, 2) seeking acceptable care, 3) physically reaching or ‘getting’ care, 4) paying for care, and 5) engaging in care (including with health care providers). Secondly, for each patient-side domain, there is a corresponding provider-side concept: 1) approachability, 2) acceptability, 3) availability and accommodation, 4) affordability, and 5) appropriateness of services (Fig. 1).

Inductive coding was conducted on an initial sample of transcripts as well as on the researcher’s (HJS) reflective memos of the interviews. Themes and sub-themes were identified under each of the domains of the Levesque framework, thereby completing an initial coding framework. This was then refined by the research team (HJS, MFH, SD, JFL) throughout coding, using an iterative process in which nodes were added, removed, grouped, moved, relabeled, redefined and confirmed by the research team. All interview transcripts were coded using NVivo 11 qualitative analysis software (QSR International).

During analysis and interpretation, it was determined that the final domain – patients’ ability to engage with their providers as well as the appropriateness of care provided – pertained mainly to the *quality of care* delivered to patients (i.e. once patients had already reached the service or provider) more so than *access* to health care and was thus conceptually distinctive from the previous four domains. It was concluded that the findings for the last domain would be better suited for separate analysis. This paper thus includes findings from the thematic analysis of data within the first four domains of the Levesque model.

## Results

### Characteristics of participants

In total, 20 primary care providers were interviewed. Initially, 23 providers were recruited, with three withdrawing from the study prior to being interviewed. The majority of the providers were trained in Australia (85%), and the clinical experience of the providers ranged from 2.5 months to 50 years (median = 14 years). The characteristics of provider participants has been outlined in Table 1.

**Table 1:** Characteristics of participating primary care providers

Participant characteristics	Number (% total or range, as indicated)
Sex	
Female	13 (65%)
Male	7 (35%)
Location of general practice	
Central and Eastern Sydney PHN	12 (60%)
South Western Sydney PHN	6 (30%)
Nepean Blue Mountains PHN	2 (10%)
Number of	
GPs	10 (50%)
PNs	7 (35%)
GP Registrars	3 (15%)
Median years working in general practice (range)	14 (2.5 months – 50 years)
Median years working at current practice (range)	6 (1 week – 30 years)
Work status	
Full time	10 (50%)
Part time	9 (45%)
Casual	1 (5%)
Australian trained	
Yes	17 (85%)
No	3 (15%)
Language of consultation	
English only	11 (55%)
English + another language	9 (45%)
Non-English languages used by provider	Spanish, Vietnamese, Mandarin, Cantonese, Malaysian, Samoan, Russian, Polish, Sign Language

Initially, 21 patient and carer participants were recruited for the study; however, one participant was excluded from the study as the patient’s main provider was revealed to be based outside of the three participating PHNs. In total, 18 patients and two carers were included in the study, encompassing a diverse range of experiences and characteristics such as age, number of years lived with experience, and the presence of a rare or unknown condition. They had been seeing the same provider for a median of 10 years (Table 2).

**Table 2:** Characteristics of participating patients and carers

Participant characteristics	Number (% total or range, as indicated)
Sex	
Female	15 (75%)
Male	5 (25%)
Location of general practice	
Central and Eastern Sydney PHN	12 (60%)
South Western Sydney PHN	6 (30%)
Nepean Blue Mountains PHN	2 (10%)
Number of	
Patients	18 (90%)
Carers	2 (10%)
Median age in years (range)	59.5 (29–88)
Median years lived with condition(s) (range)	14.5 (1–41)
Presence of rare condition(s)	
Yes	4 (20%)
No	16 (80%)
Median years seeing current GP (range)	10 (3.5 months – 21 years)
Bulk billed by GP	
Yes	13 (65%)
No	3 (15%)
Uncertain/Did not answer	4 (20%)
Recruited from patient advocacy or consumer representative organization	
Yes	11 (55%)
No	9 (45%)

### Access issues informed by patient experience of general practice

Findings are presented for each pair of domains of the Levesque framework.

### Domain one: perceiving need and approachability of care

Perceiving the need for care is often the first step in the patient journey and is determined by their individual knowledge and skills (including basic health literacy), as well as any existing beliefs about health and sickness [30]. The *approachability of health services* refers to the practice or provider’s efforts to make their services more known to their patients, and is often related to transparency, outreach, and provision of information to patient communities [30].

In our context of long-term GP attenders, approachability had less to do with outreach to new patients than the provision of information, resources, and education about services to the practice’s existing clientele. This included education about services relating to various aspects of chronic disease management – not only for GP-based services, but also for allied health and specialist services. In our study, this focused around patients’ ability to perceive the need for: 1) attending basic general practice-based services (e.g. GP visits) and 2) returning for routine visits.

#### Perceiving the need for GP-based services

Overall, the patients and carers did not report a major barrier at this point of access, given their long-term attendance at general practice. This experience appeared to give patient interviewees good knowledge of their conditions and understanding of their care needs, including the need to initiate Care Plans (Table 3, Q2). However, some providers noted that barriers remained for specific vulnerable patient groups. An example was provided by a GP registrar who observed the impact of her patients’ cultural background (potentially confounded with poor health literacy) on their ambivalence toward the importance of GP-based care, in this case for preventive services (Table 3, Q3). Another GP found that among some of the patients who are referred to his community mental health clinic, existing mental illnesses served as a barrier to attendance at his clinic (Table 3, Q4). The same GP described his solution was to establish an understanding of the benefits and services that the GP could offer them (Table 3, Q5).

**Table 3:** Perceiving the need for GP-based services

Topic	Themes and examples
Factors affecting ability to perceive need for GP-based services affecting patient sub-groups	*Perceiving overall need for regular GP* Q1 “I think especially having chronic illnesses, I’ve probably found in the past I haven’t necessarily had a regular GP and I can definitely see the benefit in having a regular GP who has an understanding and overview of your medical history, especially if you’ve had long term chronic illnesses.” (Patient 3 CES) Q2 “I think over the years the patients are more educated. They don’t ask questions anymore because they understand what [a Care Plan] is and they also find out from friends and things like that [..] now they hardly ask anything. Before they [used to ask] why am I doing this? Why do I need to do this? Now they initiate that at the consult, ‘I need to do a Care Plan.’” (GP 6 SWS) *Culture and health literacy barriers* Q3 “We have a lot of Pacific, Samoan and ethnic populations. They’re at more risk of getting certain conditions, so things like diabetes, obesity, cardiovascular disease. There is an element, whether it’s a health literacy thing or a cultural background, where they don’t necessarily understand the severity of preventative health and having problems with things that can be presented.” (GP Registrar 1 SWS) *Mental illness* Q4 “[…] some of the patients don’t show up because they don’t understand why they need to see a GP. That could be partly due to their mental illness that limits their insight into attending a GP […]” (GP 2 CES)
Offering patient education on need for GP visits	*Educating patients on need for GP visits* Q5 “[…] sometimes when the patient comes in to see me they’re not entirely sure why they’re here to see me so I have to really explain and go out of my way to explain the benefits and the services that I can offer to them as a GP. That could be anything from doing a general health check-up, physical examination, ordering some routine tests like blood tests.” (GP 2 CES)

#### Routine visits to GP

A priority expressed by several providers was to ensure routine attendance at general practice to ensure appropriate management of chronic diseases through regular review of their Care Plan (Table 4, Q1, Q2), better managed appointments (Table 4, Q5), and follow-up of complex issues (Table 4, Q4), so that patients were not “just turning up when they’re sick”. However, providers felt that not all patients prioritize or are interested in returning for appointments, for example due perceived lack of time or preference to use GP services for more acute needs (Table 4, Q1, Q2).

**Table 4:** Perceiving the need for making routine visits to GP

Topic	Themes and examples
Patient priorities and interest in routine chronic disease management	*Patient does not prioritize routine visits* Q1 “[…] sometimes we organize a follow up appointment but they may not necessarily return at that specified day or they just come when they want to” (GP 2 CES) Q2 “[…] certainly here in the affluent suburbs of Sydney, my experience of people using our service is that most people are not that interested […] they feel like they haven’t got time. They just rely on their medication. Managing that chronic disease is not a priority. They’ll come in if they’re really sick […]” (Practice nurse 8 CES)
Provider prioritizing routine care	*Provider reasons for ensuring routine care* Q3 “I think good quality of care is […] making sure they come back for regular review.” (Practice nurse 12 CES) Q4 “[…] a patient who is a regular, routine patient. They will most likely be booked in to see me because we are following up a complex problem […] monitoring something like diabetes or hyperlipidemia or checking out their bone mineral density.” (GP 5 NBM) Q5 “[…] when I came here they didn’t have any really structured chronic disease management program so not many health assessments and not many care plans. We’re slowly changing that system to look at trying to get patients to come in proactively or to be proactive in having patients come back for better managed appointments rather than just turning up when they’re sick.” (Practice nurse 9 CES)

### Domain two: seeking health care that is acceptable to patients and their carers

After establishing the need for care, the next access domain is *patients’ ability to seek care* that is acceptable to them. A*cceptability* in Levesque’s framework is described broadly as the suitability of health services to patients based on varying social and cultural needs [30]. Again, most of our patients and carers did not report experiencing major barriers in seeking acceptable care, but some key issues were still raised (Table 5).

**Table 5:** Socially, culturally, linguistically acceptable services

Topic	Themes and examples
Considerations for socially acceptable and need-based care	*Inclusion of family and carers* Q1 “[The previous GP] came to know us as a family and […] He was concerned about my husband’s condition and he was concerned about me. He saw us as a package, as a couple. He saw our conditions as individual, but we were two together and how one impacted on the other.” (Carer 4 SWS) *Gender-based preferences for GPs* Q2 “I really liked [the previous female GP], and I thought wow, I think I’d feel really comfortable having a pap smear or something with her. I don’t know that I’d feel with the other GP, the male one […] I heard a lot of people have said it would be really great if he had a female GP in the practice as well.” (Patient 1 SWS)
Considerations for culturally and linguistically acceptable care	*Catering to patient’s linguistic preferences* Q3 “So I speak Samoan. So that’s very helpful. So I tell all our medical students and doctors ‘if you have a second language please use it, because it will only be of benefit to your patients’.” (GP 11 SWS) Q4 “The receptionist can speak English and Vietnamese, because some of my patients, it’s hard to make a booking in English so they’d prefer to speak in Vietnamese, so they call us to make a booking.” (GP 6 SWS) *Working as a GP team to offer more culturally acceptable care* Q5 “So we talk about cultural competency or being medically competent. So medically competent is what our doctors are. Now they understand signs of an upper respiratory infection, but they need to understand the culture and what’s going on. So we talk about the domains of general practice and that’s something that even our medical students learn about and we learn about the one on one relationship that we deal with the patient’s demographics. Then we deal with the psycho-social environment, then we deal with the medical-legal. Then we put all that together to manage a patient in general practice.” (GP 11 SWS) *Cultural acceptability of specialist services* Q6 “[W] e do have a language barrier with the Vietnamese culture. Sometimes, especially mental health, they do have the issue and they don’t tell because of the social stigma there […] they just want to talk to you because when they talk, they worry about the information leaking, even though you explain that everything’s confidential, but they don’t trust.” (GP 6 SWS)

#### Socially, culturally, linguistically acceptable services

Among the acceptability issues that were raised were social, cultural and linguistic challenges to meeting patient needs such as gender-based preference for GPs (Table 5, Q2), sufficient involvement of carers in patient care by the GP (Table 5, Q1), and the availability of culturally and linguistically compatible providers (Table 5, Q3–6). While these issues did not appear substantial enough to prevent patients from seeking care in general practice, culture and health belief remained fairly important consideration in the perceived acceptability of services, particularly for mental health care. One provider serving mainly elderly and newly arrived Vietnamese community noted her patients’ aversion to discussing mental health issues because of the cultural and social stigma of seeking mental health care, and the fear surrounding privacy of this sensitive information (Table 5, Q6). In order to address this, the practice hired a part-time psychologist of Vietnamese background to see patients within the practice. Another GP also reported to employing new staff who spoke the most common language of the patient community, and at the same time, encouraging its existing staff to use the patient’s preferred non-English language whenever possible (Table 5, Q3). In addition to these measures, this practice also engaged in peer discussions and training junior staff on how to offer more culturally sensitive and acceptable care to their patient demographic (Table 5, Q5).

### Domain three: reaching needed care

*Ability to reach health care* pertains to the patient’s ability to physically access services, based on factors such as personal mobility (e.g. presence of disability, access to transportation) and external circumstances such as occupational flexibility. The *availability* and *accommodation* of services refer to the presence of needed health services and providers, as well as their accessibility to patients both physically and in a timely manner [30].

#### Patient’s ability to physically reach general practice

Several patients identified physical mobility as a factor in their ability to attend services. This was unsurprising as chronic conditions encompass a range of illnesses, symptoms, and disability, and depending on their severity can be debilitating to patients. Some of the challenges identified were physical disabilities (Table 6, Q2, Q5), chronic pain (Table 6, Q1, Q5) and fatigue (Table 6, Q1). In more severe cases, illness, compounded by age and frailty, prevented general practice attendance completely (Table 6, Q2). One young patient described having to choose between attending her GP appointment and completing other daily tasks due to the pain and fatigue caused by her chronic condition (Table 6, Q1).

**Table 6:** Physically reaching the general practice

Topic	Themes and examples
How patients cope with reduced mobility and other disabilities	*Reduced mobility due to disability* Q1 “Sometimes just getting up and making a meal is difficult to do. It’s painful and also difficult. You feel like you’re carrying sacks of potatoes on your shoulders, so mobility is an issue. My mobility is around fatigue-ability. I plan my appointments around other needs that I have throughout the week. So, sometimes that has impacted whether I can see him or not […]” (Patient 1 CES) Q2 “[Husband] can’t get there unless I take him, because he’s in a wheelchair. So, I have to be available to take him […] Sometimes my husband, because he’s in bed, and he’s sick, I can’t get him dressed, and get him into his chair, and get him to the doctor’s. It’s not feasible. […] This is a sick man. He’s 70. He’s got progressive incurable disease. I can’t get him to the doctor. I can’t just snap my fingers and produce him there.” (Carer 4 SWS) *Access to transport and accompaniment* Q3 “Usually, I know that I will be back in two weeks’ time so I can make the appointment then or come home and get the appointment after checking with one of my children or person that can drive me to the place.” (Patient 4 CES) Q4 “When they do come in to see me it definitely aids their attendance if someone accompanies them. So, whether it’s a family member or a case worker, either of the mental health service or from a support organization – there’s these mental health organizations that offer volunteers. Case support officers.” (GP 2 CES)
How providers and practice accommodate patient mobility needs	*Providing alternative modes of service delivery* Q5 “The other thing that I really value about my GP is that she also does home visits. If I’m having a particularly bad time with my rheumatoid, for example my mobility’s limited, she will come and do a home visit so that I don’t have to get to her […] I do know that I’ve got that as a backup if need be.” (Patient 5 CES) Q6 “we do home visits. We do clinics over the phone. We make sure that patients can access all of the services that they need to for their own sort of healthcare condition.” (Practice nurse 7 SWS)

Some patients worked around their reduced mobility by arranging transport through family and friends whenever possible (Table 6, Q3, Q4). In a more extreme example, where severe mental health illness was involved, accompaniment by carers, or even mental health care workers, helped facilitate attendance (Table 6, Q4). A number of providers and practices reported offering alternatives to accommodate patients’ various mobility-based needs (“We make sure that patients can access all of the services that they need to for their own sort of healthcare condition”). Some of the common solutions were GP home visits (Table 6, Q5, Q6) and over-the-phone clinics (Table 6, Q6).

#### Scheduling and attending routine visits with GP

For patients with a regular GP or nurse, scheduling routine visits did not appear to be a major barrier. It was typical or standard practice for subsequent or follow-up visits to be booked at the end of the consultation (Table 7, Q1). One GP remarked that pre-booking appointments was essential in the management of those with complex conditions for ensuring ongoing care, monitoring, and follow-up (Table 7, Q2). As for patients, the availability of phone or online self-booking features was useful to organize follow-up appointments. Mobile reminder and recall systems were another practical means by which practices facilitated return visits (Table 7, Q3). Whilst these scheduling tools were a common feature for many of the larger group practices, the lack of such technological features negatively impacted solo practices, which subsequently led to considerable organizational challenges for the provider (Table 7, Q4).

**Table 7:** Scheduling and attending routine visits with GP

Topic	Themes and examples
Practice features for booking return or follow-up visits	*Pre-booking follow-up appointment* Q1 “With my GP, we always book an appointment at the end of a consultation so that I’ve always got one booked when I leave, so we have a regular review. […]” (Patient 5 CES) Q2 “[Patients] will have an appointment pre-made. I have found that with chronic problems, if you basically say, “Well do this and then get back to me,” then at times people either ring up, can’t get in when they want to, and everything lapses. So, for follow-up of chronic problems, I usually pre-book the appointment.” (GP 5 NBM) *Practice features for appointment booking and recall, reminder* Q3 “[Patients] will get a reminder on their phone the day before, and the majority of our patients in our practice now do have mobile phones, so there’s very few of them that don’t get their SMS reminder. That gets sent out the day before and then if it’s a long appointment, they will actually be asked to ring in and confirm the appointment.” (GP 10 CES) Q4 “There is no booking system here […] Most practices book and I don’t […] The disadvantage is it’s actually chaotic. In other words, I can’t keep organized like the other practices. On the plus side I tend to see slightly more patients than the appointment system [would allow].” (solo GP 4 CES)
Seeing a regular provider & acceptable alternatives	*Encouraging and facilitating visits with other GPs in practice* Q5 “If you can’t get in to see him […] they would give you an appointment for one of the other doctors and he and the other doctors have said to me, ‘Don’t worry, we always confer with each other.’ If you were to see another doctor, that doctor would give him the details of what was happening, so he would be up with what was happening to you.” (Patient 8 CES) Q6 “[GPs at the clinic] are often fully booked and their patients are going “I only want to see Dr. so and so”. So we’ve got this exercise at the moment, trying to change the mindset of patients. ‘We’ve got these great doctors who have a shared medical record and so if you can’t get to see your regular doctor you can see one of these other doctors that are available so we try and facilitate that.’” (Practice nurse 9 CES)

In instances where the patient’s regular or preferred provider was not available (e.g. holidays, reduced work hours) practices encouraged and enabled continuity of care for patients within the same practice. This was typically done using a medical record system shared between providers (Table 7, Q6) or through interpersonal discussions (Table 7, Q5).

#### Scheduling and attending GP visits outside of regular appointments

The pathway in general practice for accommodating unexpected or urgent visits was less straightforward. For instance, in urban areas, these barriers included distance to the general practice clinic, the provider’s availability, and the practice’s operating hours (Table 8, Q1–3). Patients living in rural areas had fewer options for acute care visits, given the scarcity of providers in the region (Table 8, Q4, Q5).

**Table 8:** Scheduling and attending GP visits outside of regular appointments (e.g. urgent or unexpected care)

Topic	Themes and examples
Limitations in seeing regular GP outside of regular visits	*Distance to general practice clinic* Q1 “I don’t live super close […] Say for example if I am actually sick with a virus or something unexpected that aspect isn’t so convenient.” (Patient 3 CES) *Limited availability of GP for non-scheduled visits* Q2 “[My GP is] only at that particular clinic on Tuesdays and Saturdays so if I had something unexpected happen where I couldn’t forward plan I wouldn’t be able to see him at that clinic.” (Patient 3 CES) *Practice operating hours incompatible with patient schedule* Q3 “Sometimes we’ll have a client who’s working from 8:00 to 4:00 or 9:00 to 5:00. Then we won’t be able to meet their needs because some will ask for the weekend service, which we don’t have, and our clinic opens at 8:00 and closes at 4:00. That’s probably the accessibility [problem].” (Practice nurse 4 CES) *Lack of GPs in remote area* Q4 “Often I get really nervous about ringing up on the day because I’m like am I going to get an appointment or is my sickness that important? […] We should be able to have access to doctors. I think they could work on that. I think we need a lot more GPs in the mountains generally. I used to live in the city and I never had trouble getting a GP [...]” (Patient 1 NBM) Q5 “To get to the GP outside of a visit is almost impossible.” (Carer 2 NBM)
Flexibility in appointment booking and communication	*Flexible communication with patient in between visits* Q6 “I always know that I can contact her at any time in between appointments if need be. I feel very comfortable and very confident to call her or text or email her - they’re our methods of communicating - and say, ‘Things aren’t great. Can I see you sooner?’” (Patient 5 CES) Q7 “sometimes they ask for your business card. I provide it and just tell them Monday to Wednesday I’m upstairs, then Friday another nurse, [Nurse Name], is upstairs. Often we won’t be the ones to answer the call but our receptionist will write down their questions or will make sure that we received the note, so we’ll call the patient back. Sometimes we can answer their questions through the phone […]” (Practice nurse 4 CES) *Flexible appointment options (incl. Drop ins)* Q8 “I think having a service that is accessible, so having a mixture of appointments, drop-ins or emergencies. So when we do our booking schedule we only book two or three an hour to have that space.” (GP 11 SWS)

Some patients reported that their practice or provider was flexible with their communication and appointment options for unscheduled visits. Some practices described having a mixture of appointment methods to accommodate patient needs, leaving space in the provider’s schedule for possible drop-ins (Table 8, Q8). In rare cases, patients with close relationships with their regular providers were even able to contact their providers via their personal mobile number to re-schedule or create new appointments as needed (Table 8, Q6). Aside from flexible scheduling, most of the providers reported making themselves available to patients for answering questions over-the-phone, either directly or indirectly through messaging (Table 8, Q7).

#### Seeing provider once arrived at practice (waiting times and space)

The waiting times for services in general practice varied widely in our cohort of patients. While some expressed frustrations about what they perceived to be unreasonable waiting times, none identified this as a prohibitive barrier to seeing their regular or preferred GP. In fact, for the most part, patients were fairly understanding of long waiting times in general practice (Table 9, Q1). Interestingly, some patients even viewed long waiting times positively as a sign of the provider’s clinical thoroughness, or even popularity among patients (Table 9, Q2). Despite this, some patients who were unwilling to wait long hours to see their GPs found ways around this problem. Examples included checking with the reception staff ahead of time and turning up at a more ‘accurate’ appointment time. Other patients opted to book appointments at a time of day they knew to have the shortest waiting time (Table 9, Q3).

**Table 9:** Seeing provider once arrived at practice (Waiting times and space)

Topic	Themes and examples
Waiting time	*Perceived acceptability of waiting time at clinic* Q1 “There’s nothing you can do, you just have to wait. Some people do take longer than the others. It’s just a fact of life. No point getting upset about something that is beyond somebody’s control.” (Patient 4 CES) Q2 “Because of his high demand, people have to wait for so long in the waiting room because sometimes he’ll have to address so many issues, and because he is thorough, it does take a little bit more time.” (Patient 2 CES) Q3 “If we can get the first appointment in the afternoon at two o’clock, which the best for my husband as far as not having to wait for long, maybe the longest we’ve had to wait would be an hour. If, unfortunately, I can’t get that time slot and I have to get a later appointment, we’ve actually waited three and a half hours.” (Carer 2 NBM)
Waiting area	*Acceptability of waiting area for vulnerable patients* Q4 “They’re not aware that my husband can’t sit in a room full of people because he can’t process that cognitively. They don’t get that that’s too noisy for him, and they’ve got the TV going, and the phones are going.” (Carer 2 NBM) Q5 “I don’t really like the physical positioning of the place. Queuing for reception, you’re kind of in a walkway that people will be using to go in and out of the shopping center. I think that’d be the only thing.” (Patient 1 CES) Q6 “I can also from my office, see the entire waiting room so I tend to sit mostly with the door open so I can look at the activity out there and if someone looks like they’re not well or if it’s someone I recognize who I know needs something, then I can grab them.” (Practice nurse 9 CES)

Waiting areas were an important consideration particularly for vulnerable patients, such as those with more severe conditions or mental illness related co-morbidities. For these patients, noise, crowdedness, and lack of privacy adversely impacted the suitability of the waiting space (Table 9, Q4, Q5). In only one case, the provider reported to actively monitoring patients in the waiting area to ensure their well-being while waiting (Table 9, Q6).

### Domain four: paying for needed care in general practice

The fourth domain in Levesque’s model is patients’ *ability to pay for needed care* “without catastrophic expenditure of resources required for basic necessities” [30]. This capacity is impacted by patients’ access to financial resources, including insurance coverage, as well as personal circumstances such as employment and socioeconomic status. Consequently, the *affordability of health services* relates not only to the direct price of services and flexibility of payment arrangements offered by the practice, but also to other expenses such as the opportunity cost incurred in generating the means to pay for care.

#### Paying for GP-based services

Many of the patients interviewed had a regular provider who provided some form of bulk-billing, even within a typically private-billing practice. There were variations in policy by practices and providers. Most providers did so on a need basis, choosing to bulk-bill specific populations, such as concession card holders (Table 10, Q1, Q3) or patients selected by the providers at their own discretion (Table 10, Q1, Q2). Other practices reserved specific hours of the day for bulk-billing (Table 10, Q4, Q5), though this appeared to lead to significant waiting times for patients at those times. Overall, because bulk-billing was so common in our study, affordability was not a major issue for accessing general practice services for most patients and carers. However, our study included one extreme case which highlighted the financial hardships of full-time caregiving, compounded by significant out-of-pocket costs for GP consultations for her husband’s complex medical condition (Table 10, Q6). Here, the affordability barrier was quite prohibitive, in that the family was forced to choose between having certain basic necessities and needed medical care.

**Table 10:** Paying for general practice services

Topic	Themes and examples
Provider or practice’s bulk-billing policies	*Bulk-billing on a need basis* Q1 “I bulk bill anybody who has a pension card or any children, and I also bulk bill people, at my own discretion, who I think probably don’t really have enough money to see me or the working poor, I suppose, but to some extent that’s my own discretion. The reality is, despite being a private billing practice, I probably bulk bill about 75% or 80% of my consultations. So cost of seeing me isn’t probably a huge barrier for many people […]” (GP 3 CES) Q2 “I mean one thing I will say is they at least they charge most of their clientele for their services over and above the Medicare rebate or in my case and my wife’s case they don’t. They bulk bill us. I mean that is a significant help to the two of us […] When they did change the [bulk billing] system we had to go and explain to them […] “We can’t live with that increase in fees because it’s too much for us.” So, they said, “Right. Okay.” So far they are just bulk billing us.” (Patient 1 SWS) Q3 “The practice offers bulk billing if you have a pensioner concession card, but otherwise, no. I know my GP has.” (Carer 2 NBM) *Bulk-billing at specific hours* Q4 “Twice a week he bulk bills for two and a half hours, and it’s generally a lot busier at those times […] So, if it’s bulk billing you don’t book. You just turn up and it’s in order of when you come.” (Patient 1a SWS) Q5 “He bulk bills during the day and then in the evenings after 5:00 you pay a fee and then on a Saturday you also pay a fee so there’s bulk billing times and then times where you pay.” (Patient 3 CES)
Patient’s personal circumstance	*Financial repercussions of caregiving* Q6 “I’ve had to give up work to care for [my husband], and there’s no payment to support me in that […] We’ve gone from dual income […] to this very, very limited income. It’s $65 to see the GP for a standard consult, and a double consult, which we have to have is $95. Having to pay that is a lot of money. It’s just very frustrating, because you know you need the care, but you have to weigh up what you’re going to live without to be able to afford this visit. I find the financials of it very, very difficult […]” (Carer 2 NBM)

## Discussion

This study found that people living with one or more chronic conditions face a number of barriers when accessing care in Australian general practice. Based on our patient characteristics, our findings were mainly relevant to the general practice experiences of those who are seeing a long-term provider for their condition and have clear knowledge of their health and health care needs. In addition to their experiential knowledge, over half of our patients belonged to a peer support network, providing an additional source of health and health care information. Thus, the ability to perceive the need for care, which relates to health knowledge and literacy [30], was not reported by patients as being a barrier to access. Similarly, our cohort of mainly Australian-born, fluently English-speaking patients did not report any major challenges in seeking culturally and linguistically acceptable care, although some challenges specific to particular ethnic populations were reported by providers. However, some concerns were raised regarding the lack of female GPs.

The most significant and recurring challenges to access reported by patients were predominantly focused around their *ability to reach* or physically access services. There were complementary patient and provider-side issues in this regard. Patients reported illness-related disabilities, including limitations in physical mobility, chronic fatigue and pain that prevented from accessing primary care. In severe cases, these difficulties forced patients to forego or reschedule appointments. These difficulties also contributed to the perceived acceptability of waiting areas, although views on acceptable waiting times were varied. Access to resources such as transport and accompaniment to GP appointments were identified to be enablers to access. On the provider side, limitations in the *availability and accommodation* of health services to address these challenges were found to be a significant barrier. The limited availability of after-hours services was a frequently cited challenge for patients especially when making unscheduled or urgent visits. This finding is similar to that of a recent international study comparing Australian experiences of accessibility in primary health care with comparator countries (via the 2013 Commonwealth Fund International Health Policy Survey), which found that more than a quarter of Australian adults (27%) faced challenges accessing out-of-hours services [31].

Given these challenges, offering alternative modes of service delivery (e.g. over-the-phone consultations, home visits) was viewed very positively by patients and carers. The availability of GP home visits in particular is a relevant and timely topic facing Australian primary care. According to data from the Australian Bureau of Statistics, the proportion of Australians who had a GP home visit has more than doubled between the 2013–14 and 2016–17 survey cycles [32], reflecting a rising demand for this mode of service delivery. The popularity of home visits may be in part due to the lack of alternative options to access GPs outside of the traditional format. For example, though mentioned in our study, over-the-phone consultations are a non-billable service and not often done by GPs [33]. While there is some debate surrounding the overall cost and cost-effectiveness of GP home visits, it is clear that they are preferred by patients in the context of having few other options.

For most of our patients, cost for GP services was not identified to be a major barrier to accessing care. Bulk-billing of GP services was a commonly reported practice within our study – a finding consistent with previous literature [11, 34]. Issues surrounding patients’ ability to pay arose mainly in practices that selectively bulk-billed patients. In our study, providers used their own discretion when selectively bulk-billing their patients and we found a lack of clarity surrounding patient and provider factors. In literature, there appear to be a mix of factors known to potentially influence selective bulk-billing, including provider and patient attributes Provider factors driving bulk-billing decisions include being Australian-trained, rurally-based, belonging to a group practice, and having a high caseload [35]. On the other hand, providers may be more inclined to bulk-bill patients who have one or more chronic conditions and are facing difficult financial circumstances (i.e. concession card holders, access to private insurance, household income) [34]. However, it is unclear whether provider decisions to bulk-bill are mainly economically-driven, or whether other factors such as relational continuity and frequency of visits are involved. Based on these considerations, greater research effort is needed to establish clear criteria around patients’ ability to pay.

Using Levesque’s conceptual framework [30], we evaluated experiences of access broadly and from corresponding patient- and system-side dimensions, and are among a growing body of literature to use this framework [31, 36]. One limitation of this study is that while participant descriptions of various access issues seemed to broadly line up with the domains of Levesque’s framework, we did not verify with participants if this was the case, for instance, by asking participants about their own definitions or interpretations of each domain. Furthermore, this paper does not include analysis of data within the fifth and final domain in Levesque’s model relating to patient’s ability to engage with the health service and the appropriateness of care delivered by the provider. This was due to the fact that this paper was focused on access to primary care rather than the quality of clinical interactions within general practice consultations. This aspect of patient experience, which is beyond the scope of this current study focused on access to care, will be discussed in-depth in another paper.

A major limitation of our study is the possible underrepresentation of specific population group such as patients with limited English language skills, who were excluded as per the interview selection criteria. Inadvertently this may have led to the exclusion of culturally and linguistically diverse (CALD) individuals, including for example recently migrated patients and those of refugee backgrounds, who may be vulnerable to experiencing multiple compounding barriers when navigating an unfamiliar health care system [37, 38]. Additionally, none of the patient and carer participants identified as Aboriginal or Torres Strait Islander, who have distinct health needs and often face barriers to culturally appropriate and equitable access to health care in Australia [36, 39]. While we have captured some of their perspectives by proxy through provider interviews, we acknowledge that this gap may have significantly limited our insight into their unique experiences of access.

Moreover, while the inclusion of carer experiences was among the strengths of our study, they were still significantly underrepresented in our sample (*n* = 2). One possible explanation is that most patients who answered the call for interest were those who were autonomous and did not require additional caregiving to manage their conditions. Therefore, along with carer perspectives, the experiences of those with very severe or debilitating illnesses may have been underrepresented in this study. Findings including from the patient interviews, however, highlighted the important role of carers on patients’ experience of general practice, particularly in their ability to physically reach services. Our study also included a case illustrating significant financial and emotional impact of caring full-time for a patient with severe chronic conditions. In 2015, 2.7 million Australians were reported to be providing informal care [40]. The significance of informal carer support in chronic care and the various difficulties they face in caring for those with chronic conditions have been widely reported in literature [23, 28, 41, 42]. It would be important to expand on this qualitative study to include more carer perspectives, to ascertain their views on how their needs and capacities in caregiving can be better supported in general practice.

Findings from this study could contribute to the measurement and use of patient experience in Australian general practice. In this setting, patient experience data is mainly collected using commercially-provided accreditation surveys and as part of a national survey. However, research on how these surveys were developed is not publicly accessible. Furthermore, in both cases, patient experience is measured broadly from all individuals who access general practice, including those visiting infrequently or for acute needs only. There is currently a gap in evidence underpinning indicators that are specific to the experience of people managing chronic conditions in Australian general practice. This study attempts to fill this knowledge gap by providing in-depth qualitative research – considering patient, carer and provider views – on how patients access care, including their care needs and barriers, and possible areas for improvement in health services to appropriately respond to patient experiences.

## Conclusion

Using Levesque’s model, this study sought to understand the experiences of people living with a chronic condition when they access – namely, perceive need for, seek, reach, and pay for – care in general practice. The themes identified in our study may be helpful in informing a general practice-based patient experience measurement tool that is specific to the experience of those with a chronic condition. The barriers reported by patients and providers should also inform policy, especially around access to GPs outside of normal consultation hours and through alternative modes of delivery – issues that have implications for respecting patient preferences. The strength of our study is in providing a comprehensive and diverse overview of what patients, carers and primary care providers find relevant to patients’ experiences of general practice in Australia. Our study could be further strengthened by a similar study that expands to include perspectives from culturally and linguistically underrepresented patient groups and more carers.

## Additional files

Additional file 1: Primary Care Provider Interview Guide. Semi-structured interview guide for primary care provider interviewees. (PDF 116 kb)

Additional file 2: Patient and Carer Interview Guide. Semi-structured interview guide for patient and carer interviewees. (PDF 125 kb)

## Data Availability

The datasets used and analysed during the current study are available from the corresponding author on reasonable request.

## Abbreviations

CALD: Culturally and linguistically diverse
CES: Central and Eastern Sydney
GP: General practitioner
NMB: Nepean Blue Mountains
PHN: Primary Health Network
PN: Practice nurse
RACGP: Royal Australian College of General Practitioners
SWS: South Western Sydney
