# Supplementary material for: What matters to people with chronic conditions when accessing care in Australian general practice? A qualitative study of patient, carer, and provider perspectives
Source: BMC Fam Pract. 2019 Jun 10;20:79. doi: 10.1186/s12875-019-0973-0 (PMC6558875; doi:10.1186/s12875-019-0973-0)
Supplement: Supplementary file 1 — Primary Care Provider Interview Guide. Semi-structured interview guide for primary care provider interviewees. (PDF 116 kb) [file 12875_2019_973_MOESM1_ESM.pdf]

Additional File 1a. Primary Care Provider Interview Guide

These questions are about events leading up to, during, and after a typical visit with a patient.

Thinking about your patients with complex health needs, including multimorbidity,

1. Please describe a typical visit by your patients at your practice – from booking the appointment to leaving your office?

2. What usually takes place between visits?

These next questions are about your thoughts on patient experience in primary health care.

Thinking about the same patient population,

3. What aspects of patient experience would be most important for you to know about as their healthcare provider?
    - [offer examples]

4. What are the most important things to ask patients – regarding the services they received and how they were treated – if we want to get a sense of the *quality* of healthcare?
    - Prod: How would you define 'good quality' healthcare?

Regarding your practice,

5. Does your practice regularly collect feedback from patients on their experiences, as part of any quality improvement initiative?
    - If no, is this something that would be useful to your practice? To you as a GP/PN?
    - If yes, can you please describe how this is done? What tools do you use? Is this a formal or informal process (accreditation surveys vs. asking patients directly during consultation)? Is this effective and useful?
