# Supplementary material for: What matters to people with chronic conditions when accessing care in Australian general practice? A qualitative study of patient, carer, and provider perspectives
Source: BMC Fam Pract. 2019 Jun 10;20:79. doi: 10.1186/s12875-019-0973-0 (PMC6558875; doi:10.1186/s12875-019-0973-0)
Supplement: Supplementary file 2 — Patient and Carer Interview Guide. Semi-structured interview guide for patient and carer interviewees. (PDF 125 kb) [file 12875_2019_973_MOESM2_ESM.pdf]

Additional File 1b. Patient and Carer Interview Guide

1. Can you please talk me through a typical visit with your [GP or practice nurse] – from booking an appointment to leaving the clinic?
   - How easy or difficult is it to book an appointment with the clinic?
   - How easy or difficult is it to travel to the clinic?
     - Where is your GP/PN based?
   - How friendly are the reception staff at the clinic?

   Similarly, what usually happens between these visits? [prompt if not mentioned]
   - [things to ask about] screenings, tests, referrals to specialists, self-management, etc.
   - Does your GP or nurse follow-up with you after visits?
     OR Does your GP or practice nurse check up on you from time to time (at home/by phone)?

2. In general, how well do you get along with your GP or practice nurse?
   [these prompt questions should be asked if patients don't mention them in their answer]
   - How long have you been seeing this current GP/Practice Nurse? How often do you visit him/her in a year?
     - What conditions are you currently seeing your GP for?
     - How long have you had [this condition]?
     - Do you consult with your GP/PN in a language other than English?
   - Does he/she know you well as a person?
   - Is he/she easy to understand most of the time? Do they explain things carefully?
   - Do you often make decisions about your health together with your GP/PN? Is that important to you?
   - How well do they listen to any worries or concerns you might have?

3. Based on your experiences of care at the GP clinic, what are the most important areas for improvement [e.g. using simple language, cost issues, other access issues, interactions with clinic staff]?
   - Does your GP or Practice Nurse know about these areas of concern?
   - [Prompt if No] How comfortable would you feel about talking to your GP or Practice Nurse about these areas of concern?
   - *Does your GP or other staff at the clinic ask you for your feedback on your experience of care? [prompt/follow-up question]*
     - *If yes: How do they ask you for your feedback?*
     - *If no: Is this something you would find useful or important to do on a regular basis?*
   - [PROMPT if 'quality' issue not brought up] Thinking back on your answers today, what does 'good quality of care' mean to you?
